# Mitochondrial dysfunction in sepsis: nutritional strategies for restoring bioenergetic homeostasis

**DOI:** 10.3389/fcimb.2026.1878518

**Published:** 2026-08-26

**Authors:** Lirong Zheng, Xuemin Yin, Ruifen Zhang, He Su, Tingting Jia, Nannan Shan

**Affiliations:** 1 Department of Cardiology, Second Affiliated Hospital of Tianjin University of Traditional Chinese Medicine, Tianjin, China; 2 College of Acupuncture, Moxibustion and Tuina, Tianjin University of Traditional Chinese Medicine, Tianjin, China; 3 Department of Critical Care Medicine, Inner Mongolia Autonomous Region Hospital of Traditional Chinese Medicine, Hohhot, Inner Mongolia, China; 4 Department of Critical Care Medicine, Hulunbuir Zhongmeng Hospital, Hulunbuir, Inner Mongolia, China

**Keywords:** bioenergetic homeostasis, mitochondrial dysfunction, mitochondrial reactive oxygen species, mitophagy, pyruvate dehydrogenase complex, sepsis

## Abstract

**Background:**

Sepsis is increasingly recognized as a syndrome of maladaptive bioenergetic failure in which mitochondrial dysfunction—rather than being a secondary epiphenomenon—acts as a central driver of immune paralysis, endothelial incoherence, and multiple organ injury. Three interconnected molecular lesions are particularly consequential: impairment of the pyruvate dehydrogenase complex (PDC), excessive mitochondrial reactive oxygen species (mtROS) generation, and defective mitophagy. These mechanisms disrupt substrate oxidation, amplify oxidative injury, and prevent effective organelle turnover, creating a self-reinforcing bioenergetic collapse that persists despite hemodynamic stabilization. Nutritional molecules that target these specific mitochondrial nodes may offer a rational adjunctive strategy, yet their mechanistic basis and translational evidence have not been systematically integrated.

**Methods:**

This mechanism‑driven review followed a PRISMA‑structured protocol to identify studies elucidating PDC impairment, mtROS excess, and mitophagy dysfunction in sepsis, as well as the therapeutic rationale for thiamine, carnitine, and coenzyme Q10 (CoQ10). We searched PubMed, Scopus, Web of Science, Cochrane Library, and ClinicalTrials.gov for English‑language literature published between January 1, 2005 and February 1, 2026. Eligible studies addressed mitochondrial dysfunction in sepsis, reported on at least one direct mitochondrial parameter (PDC activity, mtROS, mitophagy markers, membrane potential, or ATP), and evaluated the specified nutritional interventions. Of 1,324 initially identified records, 105 studies met inclusion criteria and were qualitatively synthesized.

**Results:**

Preclinical evidence establishes that PDC impairment—driven by thiamine pyrophosphate deficiency—reduces pyruvate oxidation and increases lactate diversion, while excessive mtROS activates the NLRP3 inflammasome and amplifies inflammation, and defective mitophagy allows damaged organelles to accumulate, sustaining bioenergetic failure. These lesions propagate across immune, endothelial, parenchymal, and cerebral compartments, manifesting as immune paralysis, microcirculatory dysfunction, cardiac and renal impairment, and sepsis‑associated encephalopathy. Thiamine supplementation restores PDC activity and improves lactate clearance; L‑carnitine facilitates mitochondrial fatty acid trafficking, with post‑hoc analyses suggesting mortality benefit in patients with baseline acetylcarnitine >35 µM; and CoQ10 stabilizes electron transport, with trials reporting reduced vasopressor duration and improved SOFA scores. However, human data remain limited to small trials and subgroup analyses, and no large‑scale randomized controlled trial has definitively established mortality benefit for any of these agents.

**Conclusion:**

Mitochondrial dysfunction—specifically PDC impairment, mtROS excess, and mitophagy failure—is a core mechanistic axis of sepsis that drives bioenergetic collapse across multiple organ systems. Thiamine, carnitine, and CoQ10 target complementary nodes within this integrated damage network and are mechanistically grounded interventions, but the field is constrained by biological heterogeneity, lack of routine biomarkers, and inconsistent clinical translation. Future progress will require biomarker‑stratified trials that match intervention to dominant mitochondrial lesion, incorporate direct mitochondrial function endpoints, and test these nutrients within defined septic phenotypes, rather than as undifferentiated supplements.Overall, I believe that the inclusion of one or two well-designed original figures would significantly strengthen the manuscript and make it more engaging and accessible to readers.

## Graphical Abstract

**Figure:**
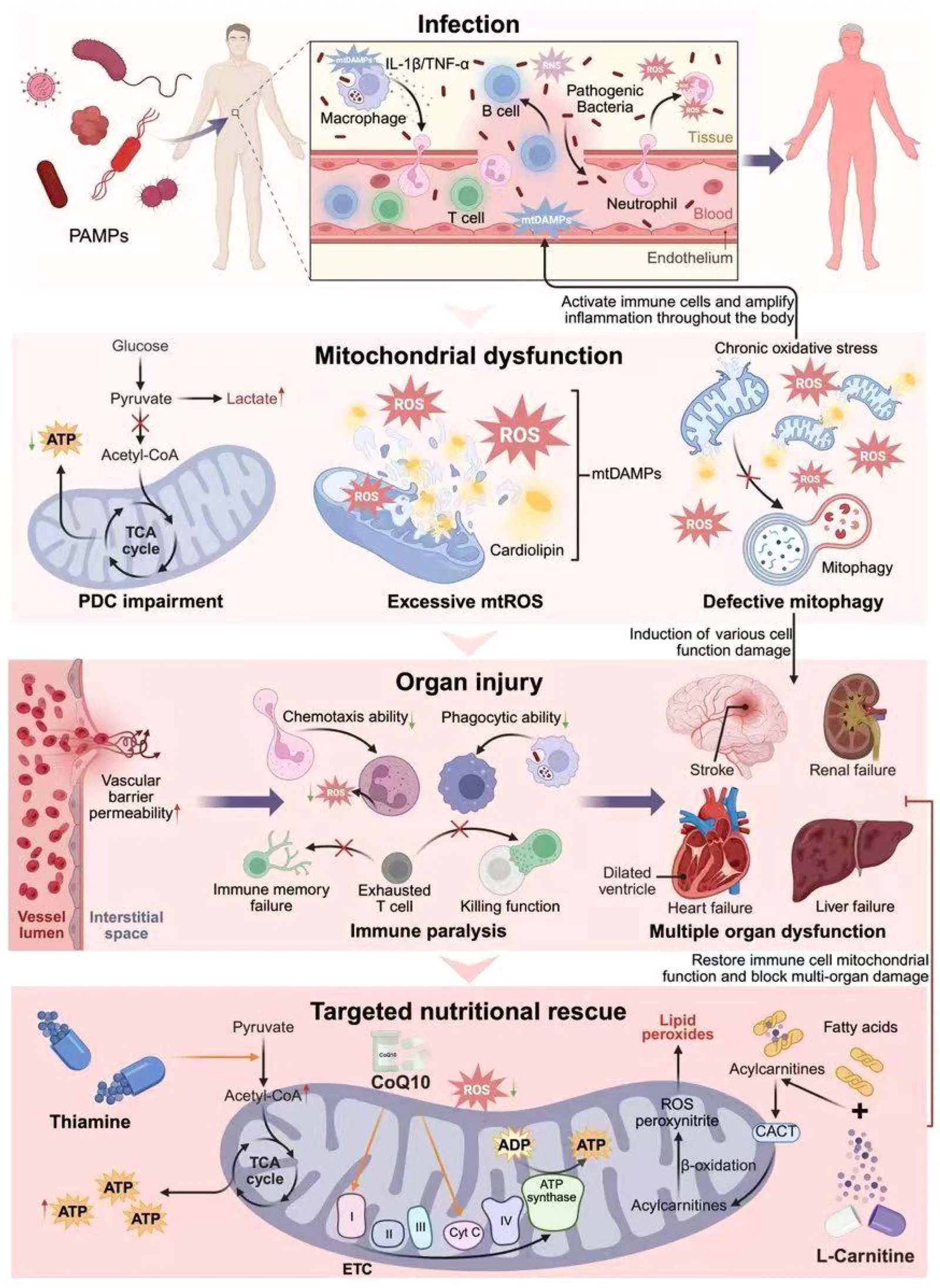

## Introduction

Sepsis remains one of the most formidable challenges in critical care medicine because it is not merely a disorder of infection, but a syndrome of dysregulated host response culminating in widespread cellular dysfunction and organ failure (Liu et al., 2024). Traditional models emphasized circulatory collapse, uncontrolled inflammation and impaired oxygen delivery as the dominant explanations for septic injury (Dobson et al., 2024). Although these processes remain important, they do not fully account for the persistent mismatch between macrocirculatory stabilization and continued organ dysfunction frequently observed in septic patients. Increasingly, sepsis is understood as a state in which cells fail not only because oxygen and substrates are insufficiently delivered, but because mitochondrial systems become unable to utilize them efficiently (Nedel et al., 2023; Wasyluk and Zwolak, 2021). This shift has repositioned mitochondrial dysfunction from a secondary epiphenomenon to a central determinant of septic pathobiology.

Mitochondria are indispensable to cellular homeostasis because they govern oxidative phosphorylation, ATP production, redox signaling, intermediary metabolism and stress adaptation (Casanova et al., 2023). In sepsis, these functions become progressively disturbed through converging molecular insults that include metabolic reprogramming, oxidative damage, calcium dysregulation, inflammatory signaling and defective organelle turnover (Liu et al., 2022). The consequence is not simply reduced energy production, but a broader state of bioenergetic disorganization in which cells are unable to match metabolic supply with functional demand. This is particularly consequential in tissues with high energetic requirements, including the heart, kidney, liver, skeletal muscle, immune system and vascular endothelium. In such tissues, mitochondrial failure contributes directly to impaired contractility, immune paralysis, endothelial instability, reduced tissue responsiveness and delayed recovery from injury.

One of the most important strengths of the mitochondrial framework is that it helps explain why organ dysfunction in sepsis often persists despite apparently adequate oxygenation, hemodynamic support and antimicrobial therapy (Srivastava and Singh, 2024). A septic patient may achieve acceptable systemic perfusion parameters yet still exhibit profound cellular hyporesponsiveness because mitochondrial enzymes, electron transport pathways and quality-control systems remain dysfunctional (Preau et al., 2021). This concept has major therapeutic implications. It suggests that successful management of sepsis may require not only infection control and supportive resuscitation, but also restoration of intracellular bioenergetic competence.

Among the molecular abnormalities implicated in septic mitochondrial dysfunction, three mechanisms have particular importance. The first is impairment of the pyruvate dehydrogenase complex, a key enzymatic gateway connecting glycolysis to mitochondrial oxidative metabolism (Jakkamsetti et al., 2025). When pyruvate dehydrogenase activity is blocked or suppressed then it will be directed away from being efficiently used by the cell in an oxidation process that produces energy (ATP), thus it contributes to lactate build-up and a decrease of the number of inputs into the TCA cycle, and as such a reduction of the cells overall ability to produce ATP (Nuyttens et al., 2024). This shift reflects more than a metabolic by-product; it represents a core disturbance in cellular fuel handling that can promote organ dysfunction even in the presence of substrate availability.

The second major mechanism is excessive mitochondrial reactive oxygen species generation. Although reactive oxygen species participate in normal signaling at controlled levels, their excess in sepsis promotes oxidative injury to lipids, proteins, DNA and mitochondrial membranes (Kumar et al., 2022). Oxidative stress contributes to the disruption of the electron transport system and thus disrupts respiratory chain function. The instability in membrane potential and increased inflammation are also secondary consequences of impaired mitochondria (Kumar et al., 2024). Thus, oxidative stress is not only an end result of sepsis. It promotes ongoing cellular damage and loss of energy production.

Mitochondrial quality control (mitophagy) also does not function properly in septic patients. Physiologically, when a cell determines that it has dysfunctional mitochondria, these will be removed and replaced by autophagy and biogenesis. However, during sepsis, this process can either be delayed, disorganized, or inadequate leading to an accumulation of dysfunctional mitochondria (Ye et al., 2025). Such persistence of injured organelles worsens bioenergetic inefficiency, increases oxidative burden and impairs the capacity of tissues to recover after inflammatory insult. The resulting failure of mitochondrial renewal is especially important in prolonged or severe sepsis, where recovery depends not only on survival of cells, but on restoration of functional organelle populations.

These mechanistic insights have stimulated interest in therapeutic strategies capable of restoring mitochondrial homeostasis. Among these, nutritional molecules are especially attractive because they target fundamental aspects of mitochondrial metabolism rather than isolated downstream inflammatory mediators. Thiamine, carnitine and coenzyme Q10 are of particular interest because each engages a distinct but complementary node in mitochondrial function (Idahor et al., 2025). Thiamine is essential for pyruvate dehydrogenase-dependent oxidative metabolism and may help redirect substrate flux away from maladaptive glycolytic accumulation (Kaźmierczak-Barańska et al., 2025). Carnitine plays a crucial role as an intermediate in mitochondrial substrate trafficking and fatty acid oxidation, which will therefore determine an organism’s metabolic flexibility and energy status (Virmani and Cirulli, 2022). CoQ10 contributes directly to electron transfer and maintaining redox balance; thus, it can be linked with both ATP production and controlling oxidative damage (Pallotti et al., 2021).

In that context, this review assesses mitochondrial failure in sepsis as a central pathway to organ injury and evaluates nutritional interventions to restore bioenergetic balance. It emphasizes alterations in pyruvate dehydrogenase complex activity, excessive mitochondrial reactive oxygen species production, and dysregulated mitophagy as key components of mitochondrial injury, and addresses the rationale for thiamine, carnitine, and coenzyme Q10 as potential metabolic modifiers. The goal is to synthesize mechanistic knowledge with evidence-based nutritional intervention strategies designed to reduce organ injury in sepsis. In that context, this review assesses mitochondrial failure in sepsis as a central pathway to organ injury and evaluates nutritional interventions to restore bioenergetic balance. It emphasizes alterations in the structure or activity of pyruvate dehydrogenase complex, enhanced production of reactive oxygen species within mitochondria, and dysregulation of mitophagy as key components of mitochondrial injury during sepsis. Then it addresses the rationale for thiamine, carnitine, and CoQ10 as potential pharmacological agents capable of protecting against mitochondrial injury. The goal is to synthesize knowledge about mechanisms underlying mitochondrial dysfunction in sepsis with evidence-based nutritional intervention strategies designed to reduce organ injury from sepsis.

## Search methodology

This mechanism‑driven review followed a PRISMA‑structured protocol to identify studies elucidating how PDC impairment, mtROS excess, and mitophagy dysfunction drive organ injury in sepsis, and how thiamine, carnitine, and CoQ10 modulate these pathways. Given the heterogeneity of the literature—spanning mechanistic, translational, and interventional studies—we adopted a narrative review format that incorporated systematic search elements rather than quantitative meta‑analysis.

We searched PubMed, Scopus, Web of Science, Cochrane Library, ClinicalTrials.gov, and preprint servers for English‑language literature published between January 1, 2005 and February 1, 2026, with emphasis on the past five years. Search terms covered four domains: sepsis (“sepsis,” “septic shock,” “critical illness”), mitochondrial dysfunction (“bioenergetic failure,” “oxidative phosphorylation,” “ATP depletion,” “mitochondrial quality control”), molecular mechanisms (“pyruvate dehydrogenase complex,” “mtROS,” “mitophagy,” “oxidative stress”), and nutritional interventions (“thiamine,” “carnitine,” “coenzyme Q10,” “metabolic resuscitation”). Boolean combinations were adapted to each database.

Three reviewers independently screened titles/abstracts and full texts; disagreements were resolved by a fourth senior reviewer. Eligible studies addressed mitochondrial dysfunction in sepsis, reported on PDC, mtROS, or mitophagy, and evaluated thiamine, carnitine, CoQ10, or comparable nutritional strategies. Mechanistic focus required measurement of at least one direct mitochondrial parameter (PDC activity, mtROS, mitophagy markers, membrane potential, or ATP). Methodologic quality was assessed using four criteria: clear methodology, adequate sample size, appropriate controls, and interpretable statistics. Studies failing any were excluded. Case reports, editorials, opinion pieces, and non‑English articles were also excluded. Of 1,324 initially identified records, 105 studies met inclusion criteria and were qualitatively synthesized.

**Figure 1:**
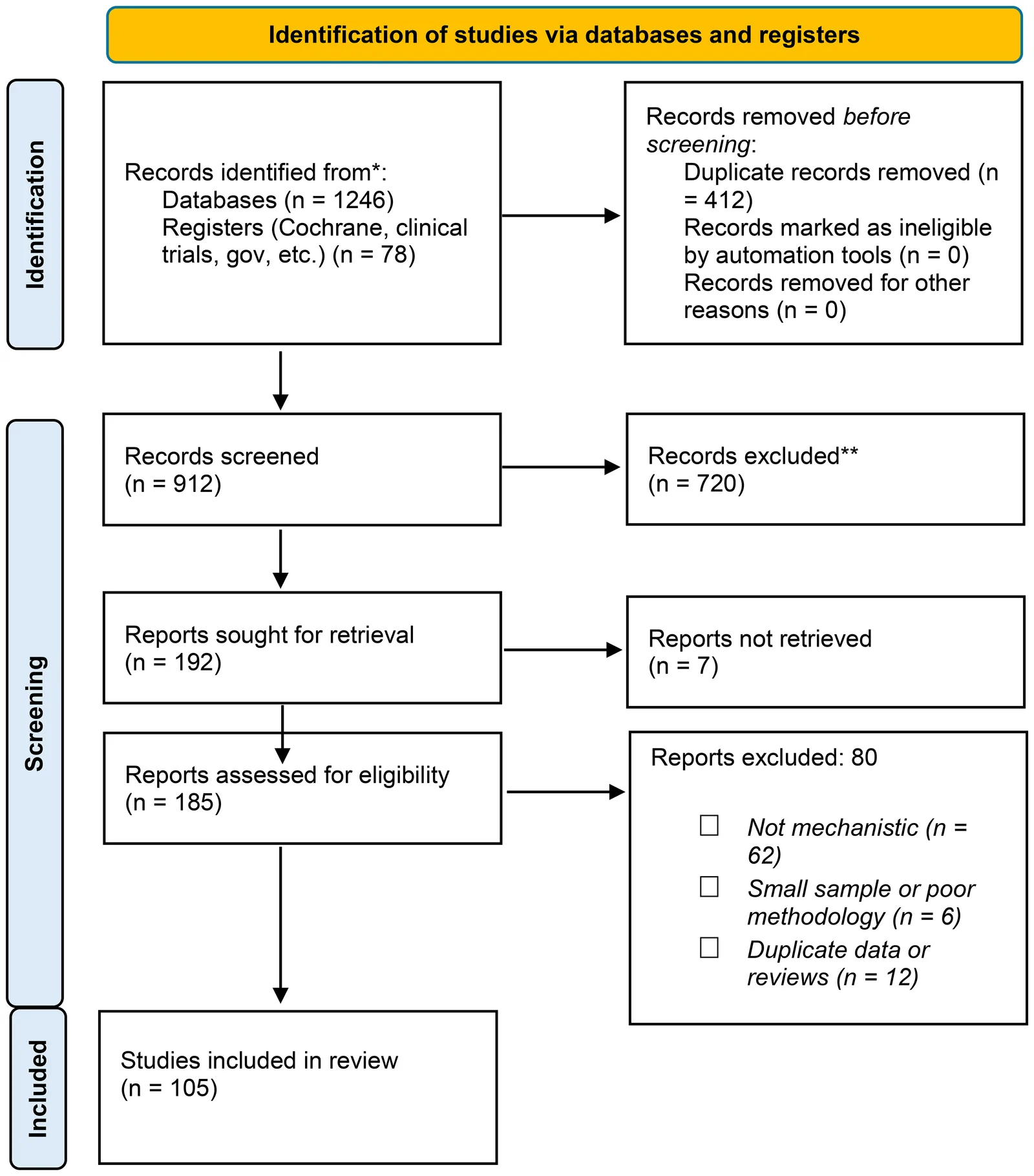
PRISMA flow chart of the included studies.

The majority of included studies were preclinical (animal or cell‑based) providing mechanistic evidence. Human observational and interventional studies were fewer and are explicitly identified when discussing clinical translation. Thus, conclusions regarding molecular mechanisms are well‑supported experimentally, whereas clinical efficacy conclusions remain hypothesis‑generating. **Table 1** summarizes inclusion/exclusion criteria, and **Figure 1** presents the PRISMA flow diagram.

**Table 1:** Inclusion and exclusion criteria for study selection.

Inclusion criteria	Exclusion criteria
Original studies examining mitochondrial dysfunction in sepsis or septic shock	Studies unrelated to sepsis or without meaningful mitochondrial relevance
Studies addressing pyruvate dehydrogenase complex dysfunction, mtROS overproduction, mitophagy impairment, mitochondrial quality control, or bioenergetic collapse	Studies lacking mechanistic focus on mitochondrial pathways
Studies investigating organ injury, immune dysfunction, endothelial damage, or metabolic failure linked to mitochondrial impairment in sepsis	Studies addressing unrelated outcomes without transferable mechanistic significance
Studies evaluating thiamine, carnitine, coenzyme Q10, or comparable nutritional mitochondrial support strategies in septic or highly relevant critical illness settings	Studies discussing nutrition without clear mitochondrial or bioenergetic relevance
Experimental, translational, observational and interventional studies with mechanistic or therapeutic significance	Case reports, editorials, opinion pieces, conference abstracts without sufficient data and narrative commentary without primary evidence
Peer-reviewed English-language publications	Non-English publications
Studies with interpretable methodology and clearly defined biological or clinical endpoints	Studies with weak methodology, unclear outcome definitions, or insufficient analytical rigor
Studies contributing directly to mechanistic synthesis or therapeutic interpretation	Duplicate datasets, duplicate publications, or review articles used only as background rather than primary evidence

## Sepsis as a syndrome of mitochondrial bioenergetic failure

### From dysregulated host response to cellular energy crisis

Sepsis is now best understood not simply as overwhelming infection, but as a maladaptive host response that converts infection into widespread cellular dysfunction (Willmann and Moita, 2024). Traditional explanatory models emphasized inflammatory excess, hemodynamic instability and impaired oxygen delivery. While these mechanisms remain important, they do not fully account for the persistence of organ dysfunction in patients whose macrocirculatory parameters have been restored. This discrepancy has shifted attention toward the intracellular level, where the septic response appears to induce a state of metabolic paralysis in which cells can no longer convert available substrates and oxygen into functional energy with sufficient efficiency (Pravda, 2021).

This transition from dysregulated host response to cellular energy crisis is driven by converging inflammatory, redox and metabolic stresses. Cellular processes are impaired as a result of cytokine signaling, nitric oxide overproduction, calcium deregulation, oxidative damage and inhibition of enzymes that reside within mitochondria (Joffre and Hellman, 2021). Cells, therefore, experience a condition with limited energetic capabilities defined by decreased availability of ATP, shifts in preferred substrates for energy and increased utilization of less efficient mechanisms to generate energy (Almalki, 2021). Importantly, this is not merely a biochemical curiosity. In sepsis, the inability of cells to maintain bioenergetic competence directly influences contractility, membrane transport, immune responsiveness, barrier integrity and tissue recovery.

### Mitochondrial injury as a determinant of organ dysfunction in sepsis

Mitochondria sit at the center of this bioenergetic failure because they regulate oxidative phosphorylation, ATP generation, redox signaling and metabolic flexibility(5). In sepsis, mitochondrial injury is not confined to one isolated pathway. It includes suppression of key metabolic enzymes, disruption of electron transport, excessive production of reactive oxygen species, membrane potential instability and failure of quality-control mechanisms that normally remove damaged organelles (Galli et al., 2024). The result is a population of mitochondria that are less efficient, more oxidative and increasingly unable to sustain cellular work.

This dysfunction has direct implications for organ injury. In the heart, reduced mitochondrial efficiency contributes to impaired contractile performance and diminished energetic reserve. In kidneys tubular cells lose their ability for sustained active transport and as a result fail in supporting cell structure (Pavlović et al., 2025). Liver function is impaired due to its inability to perform its role in metabolism and detoxification (Guo et al., 2025). Muscle atrophy occurs in muscles that contain dysfunctional mitochondria which leads to decreased strength and reduced rate of recovery (Chen et al., 2022). These organ level manifestations are not simply downstream effects of systemic inflammation; they reflect the fact that septic stress has penetrated to the level of intracellular power generation.

A key strength of the mitochondrial model is that it provides a common mechanistic language for otherwise diverse organ failures. Rather than viewing septic cardiomyopathy, acute kidney injury, endothelial dysfunction and immune paralysis as separate complications, the model suggests that they share a core pathogenic substrate: impaired mitochondrial homeostasis. This does not mean every organ fails through identical pathways, but it does mean that mitochondrial injury may be one of the most coherent unifying determinants of why multiple organ systems become hyporesponsive during sepsis.

### Bioenergetic maladaptation across immune, endothelial and parenchymal cells

One of the defining features of septic mitochondrial dysfunction is that it affects multiple cell populations simultaneously, though not identically (Chen et al., 2023). The bioenergetic response of immune cells, endothelial cells and parenchymal tissues differs according to functional demand, metabolic phenotype and exposure to inflammatory stress (Ahola, 2024; Ostojic, 2025). This cell type diversity is important because sepsis is not only a disease of organ failure, but also a disorder of failed coordination among defense, circulation and tissue maintenance.

In immune cells, bioenergetic maladaptation contributes to the paradoxical coexistence of inflammatory activation and immune inefficiency. Early in sepsis, immune cells may undergo intense metabolic reprogramming that supports inflammatory signaling but is difficult to sustain (Xian et al., 2025). Mitochondrial function is also affected by stress and as such, will result in a failure of the system to clear pathogens appropriately and subsequently, an increase in susceptibility for subsequent infections; this will result in an inability of the immune system to respond appropriately (Melis, 2022). Thus, it is likely that mitochondrial dysfunction will lead to either too much inflammation causing tissue destruction or less response from the immune system (immunoparalysis).

Disturbances in mitochondria in endothelial cells are not limited to a reduction in energy production (ATP) as they contribute to alterations in vascular tone, endothelial cell barrier function, regulation of microvascular flow and oxygen delivery to tissues (Vincent et al., 2021). When endothelial bioenergetics become compromised, perfusion becomes less efficient at the microvascular level even if systemic hemodynamic support appears adequate (Roy and Secomb, 2021). This contributes to the familiar septic pattern in which perfusion and tissue responsiveness become uncoupled.

Parenchymal cells experience a different but equally consequential form of maladaptation. Their energetic burden is tied directly to organ specific work, such as contraction, filtration, synthesis, transport and repair. Mitochondrial injury in these cells reduces functional capacity while also limiting their ability to recover from inflammatory and oxidative insult (Chen and Tang, 2022). The result is not simply reduced performance, but persistence of a low-energy state in which recovery pathways themselves are impaired. This broad cellular pattern reinforces the idea that septic organ failure is not just inflammatory injury expressed locally, but a systems-level disorder of disrupted energy handling.

### Why mitochondrial homeostasis is a therapeutic priority in sepsis

If mitochondrial dysfunction is central to septic pathobiology, then restoration of mitochondrial homeostasis becomes a logical therapeutic priority (Mokhtari et al., 2022). This does not imply that mitochondrial support can replace antimicrobial therapy, source control, fluid resuscitation, or organ support. Rather, it suggests that these measures may be incomplete if the intracellular machinery required for effective energy use remains impaired. A patient may survive the initial insult yet continue to deteriorate biologically if mitochondrial networks are unable to recover.

Mitochondrial homeostasis matters because it integrates several essential functions at once: substrate oxidation, ATP generation, redox balance, signaling control and organelle turnover (Li et al., 2024). Therapeutic strategies that stabilize these processes could theoretically improve tissue responsiveness, reduce secondary oxidative injury, support immune competence and accelerate recovery of stressed organs. This is particularly important in sepsis, where the failure to reestablish metabolic order may prolong organ dysfunction even after infection control has begun (Liu et al., 2022). A mitochondria centered therapeutic perspective also opens a more precise approach to nutritional and metabolic intervention. Rather than viewing nutritional support solely in terms of calorie provision, this framework asks whether specific molecules can correct identifiable metabolic bottlenecks, protect organelles from oxidative collapse, or improve the quality of mitochondrial recovery. Such thinking is especially relevant to thiamine, carnitine and coenzyme Q10, which target different but complementary aspects of mitochondrial function.

We do not propose mitochondrial dysfunction as a replacement for other pathogenic pathways such as immune dysregulation or coagulopathy. Rather, mitochondrial-targeted nutritional strategies should be viewed as complementary adjuncts to—not substitutes for—standard sepsis care. The rationale for highlighting mitochondrial dysfunction is to address a comparatively neglected domain that may explain persistent organ dysfunction despite adequate conventional therapy.

## Core molecular mechanisms of mitochondrial dysfunction in sepsis

### Pyruvate dehydrogenase complex impairment and disrupted oxidative metabolism

Among the earliest and most consequential metabolic abnormalities in sepsis is impairment of the pyruvate dehydrogenase complex (PDC), the enzymatic gateway that links cytosolic glycolysis to mitochondrial oxidative metabolism (Nuyttens et al., 2024). Under normal physiological circumstances, Pyruvate Dehydrogenase Complex (PDC) catalyzes conversion of pyruvate to Acetyl-CoA; thus, facilitating access into the TCA cycle with subsequent production of ATP via oxidative phosphorylation (Kumar and Greenberg, 2025). However, under conditions of sepsis, the gateway for these processes is physiologically limited; subsequently, the shift in cellular metabolism towards an inefficient energetic state results (Liu et al., 2023).

These consequences have several significant effects. Firstly, as a result of this limitation, there is increased diversion of pyruvate, unable to be metabolized within mitochondria, to lactate formation; therefore, further decreasing efficiency of metabolism, regardless of whether or not O2 delivery is at critical levels (McCall et al., 2022). Secondly, decreased availability of Acetyl-CoA severely restricts TCA cycle enzyme activity; thus, diminishing the amount of reducing equivalents delivered to the electron transport chain (Zeng et al., 2021). Lastly, alterations in oxidative handling of substrates alter how cells select their fuels; resulting in many cases where cells are placed in a condition of maladaptation whereby they are unable to meet organ level energy needs by compensating with glycolysis (Muniz-Santos et al., 2023). The consequence is not merely lactate accumulation, but a deeper failure of bioenergetic integration.

PDC impairment also has pathophysiological significance because it links inflammatory signaling to mitochondrial failure. The septic milieu favors enzyme inhibition through inflammatory mediators, redox stress and altered kinase–phosphatase balance, thereby converting host response into metabolic blockade (Lazzaro et al., 2022). In tissues with high energetic requirements, this bottleneck can rapidly impair functional reserve. In an immune cell, this may lead to misaligned metabolic programs that favor dysregulation as opposed to proper coordination of the host’s response in sepsis (She et al., 2022). The impairment also limits the ability for parenchyma to recover from sepsis through lessening the effectiveness at which available substrate is converted into usable energy (McCall et al., 2022). The mechanistic basis for this impairment has been directly established in preclinical models. Using a cecal ligation and puncture model, Nuyttens et al. demonstrated that mitochondrial pyruvate-driven respiration is nearly absent in septic liver mitochondria, attributable specifically to functional PDC failure rather than to defects in pyruvate uptake or carboxylation. Importantly, this dysfunction was traced to a shortage of the PDC cofactor thiamine pyrophosphate (TPP), as TPP supplementation restored pyruvate oxidation and protected mice from sepsis-induced lethality (Nuyttens et al., 2025). Collectively, these data indicate that PDC impairment in sepsis is a dynamic, enzyme-activity-driven phenomenon amenable to metabolic intervention.

### Excessive mitochondrial reactive oxygen species generation

Mitochondrial reactive oxygen species are integral to normal signaling when tightly regulated, but in sepsis they exceed physiological thresholds and become potent mediators of organelle injury (Itagaki et al., 2021). Excessive mtROS production arises when the electron transport chain becomes unstable, electron leakage increases and antioxidant buffering systems fail to keep pace with oxidative burden (Chenna et al., 2022; Napolitano et al., 2021). This creates a condition of redox collapse in which signaling oxidants are transformed into persistent molecular stressors.

The consequences of excessive mtROS are broad and self-amplifying. Oxidative damage to mitochondrial lipids, respiratory chain proteins and mitochondrial DNA reduces organelle efficiency and increases the probability of further electron leakage (Li et al., 2023). The mtROS also amplifies inflammatory signaling, perturbs calcium handling and destabilizes membrane integrity (Rius-Pérez et al., 2023). As oxidative burden accumulates, mitochondria become less capable of maintaining efficient ATP synthesis and more prone to signaling states that favor injury rather than adaptation (Eyenga et al., 2022).

What makes mtROS especially important in sepsis is its role as both cause and consequence of mitochondrial dysfunction. PDC impairment, respiratory chain inefficiency and membrane instability all promote oxidative excess, while oxidative excess further worsens each of those abnormalities (Joffre and Hellman, 2021). The sources of this oxidative excess have been traced to specific sites within the electron transport chain, particularly complexes I and III. Mechanistically, excessive mtROS directly activates the NLRP3 inflammasome by serving as a critical “second signal” for inflammasome assembly, creating a feed-forward loop that amplifies both inflammation and mitochondrial injury. This has been confirmed in CLP and LPS-challenged models, where inhibition of mtROS production attenuates both inflammasome activation and tissue damage (Hu et al., 2024). This creates a pathogenic loop in which redox imbalance sustains energetic decline. In endothelial cells, such stress may impair vascular responsiveness and barrier control. In immune cells, it may contribute to dysfunctional activation or exhaustion (He et al., 2021). In parenchymal cells, it increases the likelihood that reversible metabolic stress will become structural damage (Chen and Tang, 2022). Redox collapse therefore represents a central point at which mitochondrial dysfunction becomes self-propagating.

### Mitophagy dysfunction and failure of mitochondrial quality control

A healthy mitochondrial network depends not only on efficient energy production, but also on continuous quality control. Damaged mitochondria must be identified, segregated and removed through mitophagy, while new organelles must be generated through coordinated biogenesis (Uoselis et al., 2023). In sepsis, this quality-control system often becomes inadequate, mistimed, or disordered. The result is accumulation of dysfunctional mitochondria that continue to produce insufficient ATP, excessive oxidants and stress signals that further destabilize the cell (Ferrer and Iba, 2024).

Mitophagy dysfunction is particularly important because it converts transient injury into persistent energetic incompetence (Zhu et al., 2021). A cell may tolerate a degree of mitochondrial damage if organelle turnover remains efficient. However, once damaged mitochondria are not removed or eliminated from a cell appropriately, they continue to be metabolically expensive, as well as dangerous (Onishi and Okamoto, 2021). The continued presence of these damaged mitochondria continues to increase oxidative burden; disrupts metabolic coordination; and decreases the number of good quality mitochondria that can assist with recovery processes (Liao et al., 2022). In particular this represents an issue during the state of sepsis, in which repeated inflammatory and oxidative stress events continuously challenge organelle integrity. To understand why mitophagy fails in sepsis, it is necessary to consider the specific molecular pathways that govern this process. Under physiological conditions, mitophagy is primarily regulated by the PINK1-Parkin pathway and receptor-mediated pathways involving FUNDC1, BNIP3, and NIX. Depolarized mitochondria stabilize PINK1 on the outer membrane, recruiting Parkin to ubiquitinate mitochondrial surface proteins and signal autophagosomal engulfment (Nuyttens et al., 2025). In sepsis, however, this system is disrupted. Gao et al. demonstrated that cellular FLICE-inhibitory protein (c-FLIP) regulates FUNDC1-mediated mitophagy during sepsis-induced myocardial dysfunction: loss of c-FLIP in early sepsis causes excessive mitophagy, while its suppression at later stages leads to accumulation of damaged mitochondria (Friedrich and Kappert, 2025). These findings establish that mitophagy impairment in sepsis is an actively regulated process driven by inflammatory signaling.

From a mechanistic standpoint, mitophagy dysfunction also links several other mitochondrial abnormalities. Oxidative injury increases the burden of damaged organelles, membrane instability impairs mitochondrial fitness and ATP depletion weakens recovery processes. When mitophagy fails in this context, the entire energetic system becomes less adaptable. Quality control is therefore not an accessory pathway; it is one of the main determinants of whether septic mitochondrial injury remains reversible or progresses toward sustained organ dysfunction.

### Membrane potential instability, ATP depletion and metabolic inflexibility

Mitochondrial membrane potential is essential for ATP production because it provides the electrochemical gradient required for oxidative phosphorylation (Morse et al., 2024). In sepsis, this gradient frequently becomes unstable as respiratory chain dysfunction, oxidative stress, calcium imbalance and membrane injury interfere with proton handling and electron transport (Ahmad et al., 2025). Once membrane potential is compromised, ATP generation becomes progressively inefficient and the cell enters a state of energy insecurity.

ATP depletion is a defining consequence of this instability. High demand tissues depend on continuous ATP availability to support transport, contraction, synthesis and signaling (Santana and Earley, 2026). When mitochondrial ATP output falls, the effects are immediate and functionally significant: myocardial performance declines, renal tubular transport fails, hepatocellular metabolism becomes impaired, endothelial stability weakens and immune responses lose coordination. Importantly, ATP depletion in sepsis is not always absolute; in some cases, it is a failure to increase production appropriately under stress (Pravda, 2021). Even this relative insufficiency can be enough to compromise organ performance when energy demand remains high.

Membrane potential instability also contributes to metabolic inflexibility. Healthy cells adapt to changing substrate availability by shifting between carbohydrate, fatty acid and other fuels while preserving mitochondrial efficiency (Alan and Scorrano, 2022). Septic mitochondria lose much of this flexibility. With PDC impairment, redox stress and defective organelle quality control, cells become increasingly unable to transition smoothly between metabolic programs (Kim et al., 2023). Instead, they remain trapped in inefficient substrate handling patterns that favor energy deficit and by product accumulation over adaptive fuel use. This inflexibility is especially damaging in sepsis where cellular demands are dynamic and rapidly changing.

Thus, membrane potential instability and ATP depletion should not be viewed as late-stage findings alone. They are functional expressions of the broader mitochondrial crisis and major contributors to the inability of organs to recover despite systemic support.

### Integrated mitochondrial damage networks driving septic organ injury

The mechanisms described above are analytically distinct but biologically inseparable. PDC impairment reduces efficient substrate entry into mitochondrial oxidation. Respiratory instability and disrupted substrate flow increase mtROS production (Jana and Alayash, 2025). Excessive mtROS damages membranes, enzymes and mitochondrial DNA, worsening energetic inefficiency (Napolitano et al., 2021). Mitophagy dysfunction prevents effective clearance of damaged organelles, allowing low performance mitochondria to accumulate (Zhu et al., 2021). Membrane potential collapses, ATP production falls and cells lose metabolic flexibility. Together, these processes form an integrated damage network rather than a sequence of isolated events.

This integrated model is essential for understanding septic organ injury. A heart cell, renal tubular cell, endothelial cell, or immune cell may not fail because of one dominant mitochondrial defect, but because several interlocking abnormalities cross a functional threshold simultaneously. Organ dysfunction emerges when energetic insufficiency, redox stress and defective organelle turnover converge in a tissue already burdened by inflammatory challenge (Toro-Pérez and Rodrigo, 2021). This integrated damage network is not confined to the mitochondrial compartment but radiates outward to propagate systemic inflammation and organ-level decompensation. When mitochondria become dysfunctional due to PDC impairment, respiratory chain instability, and defective mitophagy, the resulting bioenergetic failure and redox imbalance trigger the release of mitochondrial danger signals—including mtROS, oxidized mtDNA, and cardiolipin—into the cytosol and circulation. These signals engage pattern recognition receptors such as NLRP3, cGAS-STING, and TLR9, thereby converting an intracellular bioenergetic crisis into a self-sustaining inflammatory response that amplifies tissue injury across organ systems. This inflammatory amplification, in turn, feeds back to further suppress PDC activity, increase electron leakage, and overwhelm mitochondrial quality-control mechanisms, creating a bidirectional crosstalk between mitochondrial dysfunction and systemic inflammation. At the organ level, this cascade manifests as the clinically familiar phenotype of sepsis: immune cells become metabolically paralyzed and lose pathogen-clearing capacity; endothelial cells exhibit barrier disruption and microcirculatory incoherence; and parenchymal tissues—heart, kidney, liver, skeletal muscle—experience functional decline that persists despite systemic hemodynamic stabilization. Thus, the molecular lesions described above—PDC blockade, mtROS excess, and mitophagy failure—are not isolated biochemical abnormalities; they are the subcellular drivers of a multiorgan syndrome in which bioenergetic incompetence sustains inflammation, inflammation deepens mitochondrial injury, and the net effect is a self-propagating cycle of organ dysfunction. This systems-level understanding directly informs the therapeutic rationale that follows: rather than targeting downstream inflammatory mediators alone, metabolic rescue strategies that relieve PDC blockade, restore substrate trafficking, and stabilize respiratory chain redox balance address the upstream bioenergetic lesions that initiate and perpetuate this pathogenic cascade. By intervening at these mitochondrial nodes, it may be possible to attenuate both the inflammatory amplification and the organ-level consequences, thereby breaking the cycle at its bioenergetic root.

This is why mitochondrial dysfunction in sepsis is best conceptualized as a systems-level process: it is distributed across pathways, sustained by feedback loops and expressed through multiple organ phenotypes. Critically, the interconnections among these pathways are not merely correlative but causally established. Experimental evidence has shown that PDC impairment increases electron leakage and mtROS production; that mtROS-induced oxidative damage further impairs respiratory chain function and lowers the threshold for mitophagy failure; and that defective mitophagy, in turn, prolongs the presence of ROS-generating damaged organelles, sustaining redox stress and ATP depletion. This self-reinforcing network has been validated across multiple tissues using both LPS and CLP models (Hu et al., 2024).

The translational importance of this network view is equally significant. If mitochondrial injury in sepsis is integrated, then therapeutic rescue is unlikely to succeed through single-node thinking alone. Nutritional or metabolic interventions must be evaluated in terms of where they act within this network and whether they can relieve key bioenergetic bottlenecks, reduce oxidative amplification, or improve organelle quality control. Understanding the architecture of mitochondrial damage is therefore a prerequisite for rational metabolic therapy.

The table below summarizes the core molecular mechanisms discussed in this section and highlights how each contributes to bioenergetic failure and organ injury in sepsis, as shown in **Table 2**.

**Table 2:** Core molecular mechanisms of mitochondrial dysfunction in sepsis: pathways, bioenergetic consequences and implications for organ injury.

Core mechanism	Principal pathway disturbance	Bioenergetic consequence	Functional implication for septic organ injury
Pyruvate dehydrogenase complex impairment (Zeng et al., 2021)	Reduced conversion of pyruvate to acetyl-CoA	Decreased oxidative substrate flux, increased lactate diversion, reduced tricarboxylic acid cycle input	Energetic inefficiency, impaired metabolic integration, reduced organ recovery capacity
Excessive mtROS generation (Chenna et al., 2022)	Electron leakage and oxidative amplification within mitochondria	Oxidative damage to proteins, lipids, DNA and respiratory chain components	Redox collapse, inflammatory amplification, worsening mitochondrial failure
Mitophagy dysfunction (Zhu et al., 2021)	Impaired removal of damaged mitochondria	Accumulation of low-efficiency, stress-generating organelles	Persistent bioenergetic dysfunction, delayed recovery, prolonged organ vulnerability
Membrane potential instability (Ahmad et al., 2025)	Loss of electrochemical gradient required for oxidative phosphorylation	Reduced ATP synthesis efficiency and impaired respiratory coupling	Functional decline in high-energy tissues, cellular hyporesponsiveness
Integrated rescue	PDC + mtROS + mitophagy concurrently	Restored oxidative flux, redox balance, organelle turnover	Reduced inflammation and improved organ function

**Figure 2:**
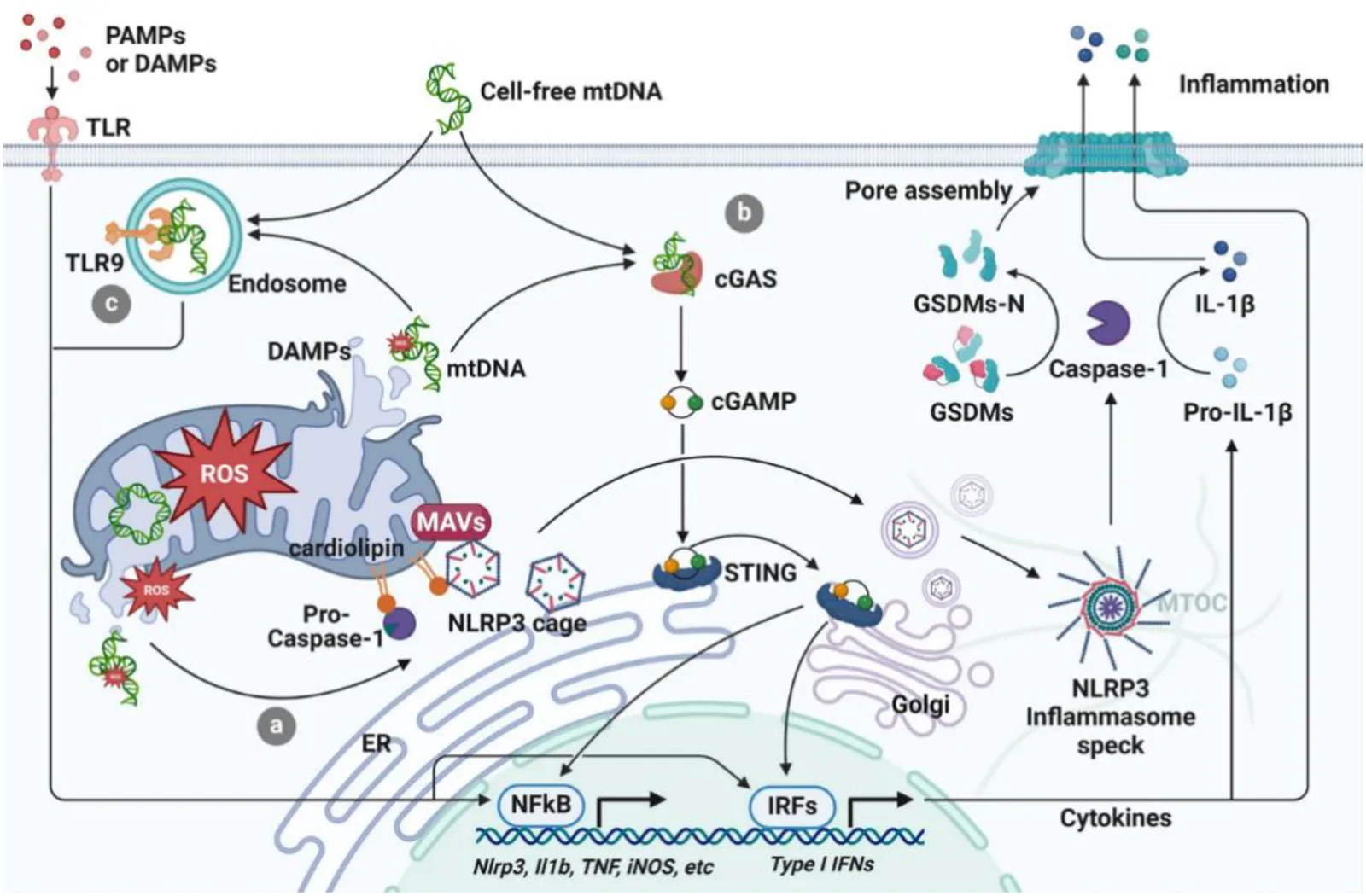
Mitochondria-regulated inflammatory pathways in sepsis. Pattern recognition receptors (PRRs) of immune cells recognize pathogen-associated molecular patterns (PAMPs) from microbes or damage-associated molecular patterns (DAMPs) released by damaged host cells. **(a)** Mitochondria orchestrate the activation of the NLRP3 inflammasome. mtROS and ox-mtDNA promote the NLRP3 inflammasome activation after their release from the damaged mitochondria. Additionally, mitochondrial antiviral-signaling protein (MAVS) and cardiolipin mediate the spatial association between mitochondria and NLRP3, which may facilitate a rapid and efficient sensing of the inflammatory signals from the damaged mitochondria by NLRP3. Early studies posited mitochondria or mitochondria-associated membrane (MAM) as the docking sites of NLRP3, while more recent publications have indicated the involvement of Golgi and microtubule-organizing center (MTOC). It could be plausible that NLRP3 proteins are first transported to meet mitochondria, after which NLRP3 redistributes to the adjacent Golgi apparatus as oligomeric cages. Then, the NLRP3 cages are transported to MTOC and reorganized into a single inflammasome speck. Activated NLRP3 inflammasome mediates the activation of pro-caspase-1, which proteolytically processes GSDMs and pro-inflammatory cytokines. The matured cytokines are released from the GSDMs pores to transmit inflammatory signals. **(b)** Mitochondria regulate the cyclic GMP-AMP synthase (cGAS)-stimulator of interferon genes (STING) pathway. cGAS is activated upon mtDNA binding, leading to the synthesis of cyclic GMP-AMP (cGAMP). cGAMP then induces a conformational change in STING, leading to its activation at ER. Activated STING translocates to Golgi, where it activates transcription factors interferon regulatory factor 3 (IRF3) and nuclear factor κB (NF-κB) to initiate the production of type I interferons and other pro-inflammatory cytokines that are required for effective immune response against pathogens. **(c)** Mitochondria regulate the TLR9 pathway. mtDNA shares similarities with bacterial DNA, thus can be recognized by TLR9. As with other TLRs and cGAS-STING axis, TLR9 pathway activates IRF3 and NF-κB to regulate the inflammatory response (Hu et al., 2024).

**Figure 3:**
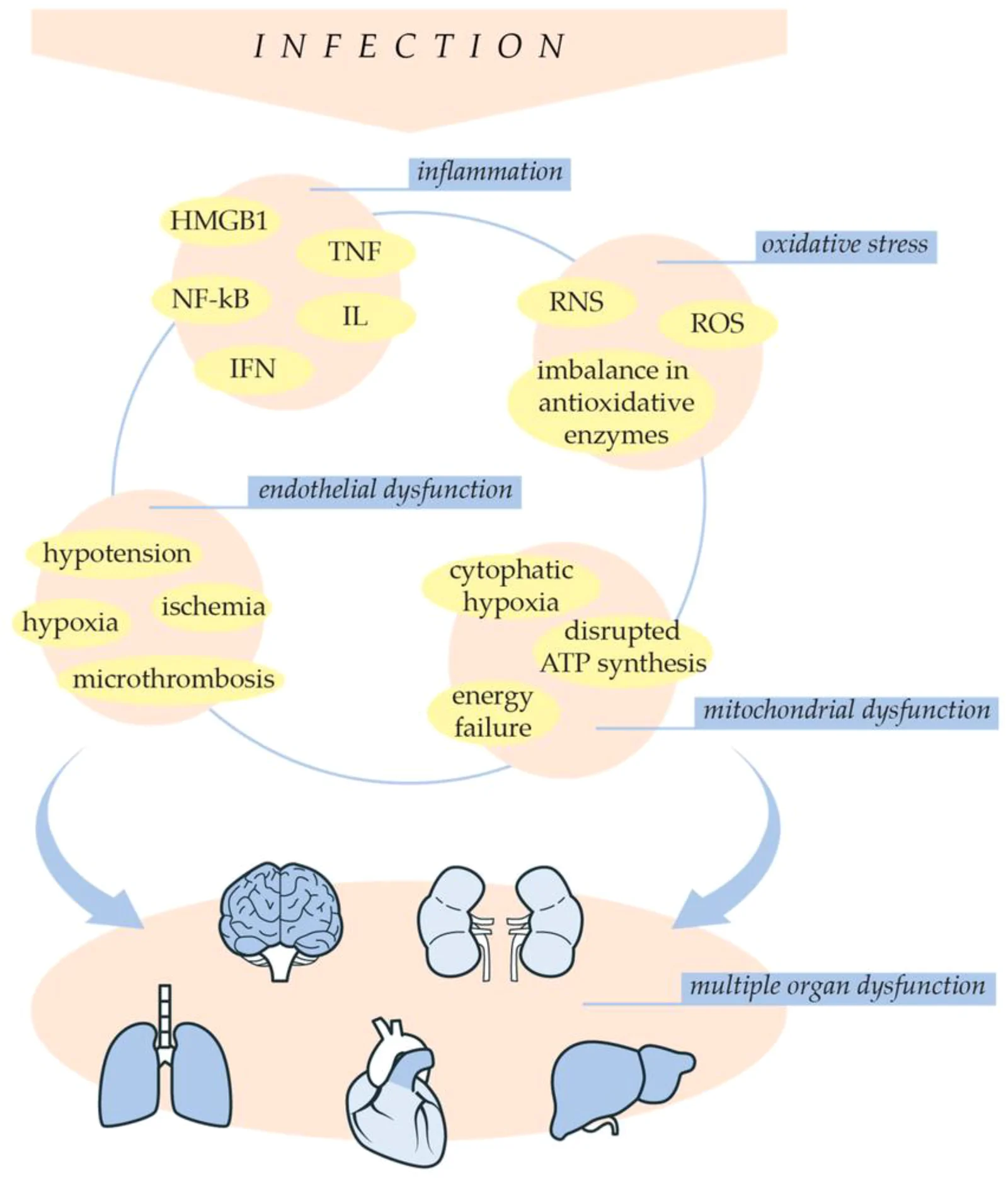
Sepsis-related multiple organ dysfunction. In response to infection, developing inflammation, oxidative stress, endothelial dysfunction and mitochondrial dysfunction can result in sepsis. Pathophysiological changes are a consequence of the cause–effect link between these processes. Together, they lead to organ damage that can progress to multiple organ dysfunction. HMGB1, high-mobility group box-1; NF-kB, nuclear factor-kappa B; TNF, tumor necrosis factor; IL, interleukins; IFN, interferons; RNS, reactive nitrogen species; ROS, reactive oxygen species (Srdić et al., 2024).

Before moving to the organ- and cell-specific consequences of these processes, it is useful to visualize the two interconnected dimensions of this pathogenic cascade. The first dimension concerns the molecular interface between mitochondrial injury and systemic inflammation: as **Figure 2** depicted, dysfunctional mitochondria release danger signals that activate NLRP3, cGAS-STING, and TLR9 pathways, propagating inflammation that further impairs mitochondrial function. The second dimension, shown in the subsequent **Figure 3**, traces these inflammatory and bioenergetic insults to their organ-level consequences—immune paralysis, endothelial and microcirculatory failure, and parenchymal dysfunction—which collectively constitute the clinical phenotype of multiple organ failure. When viewed in sequence, these two figures illustrate the complete trajectory from infection-triggered mitochondrial lesions (PDC impairment, mtROS excess, mitophagy failure) through inflammatory amplification to organ injury, and finally to the therapeutic interception points where nutritional molecules (thiamine, carnitine, CoQ10) can restore bioenergetic homeostasis.

## Pathophysiological consequences of mitochondrial dysfunction in sepsis

### Immune-cell metabolic paralysis and dysregulated host defense

One of the most clinically important consequences of mitochondrial dysfunction in sepsis is the emergence of immune cell metabolic paralysis. Effective host defense requires immune cells to adjust energy production rapidly in response to infection, inflammatory signaling and tissue injury (Rankin and Artis, 2018). In sepsis, however mitochondrial pathways become unable to sustain this adaptive flexibility. Early activation may initially be accompanied by high metabolic demand and inflammatory signaling, but persistent mitochondrial stress eventually reduces ATP generation, redox balance and substrate utilization (Griffiths et al., 2017). As a result, immune cells may enter a state in which they remain phenotypically activated yet functionally inefficient.

This dysfunction has major implications for pathogen control. Neutrophils, monocytes, macrophages and lymphocytes all depend on coordinated metabolic programming to support chemotaxis, phagocytosis, cytokine regulation, microbial killing and survival (Marelli-Berg and Jangani, 2018). When mitochondrial performance declines, these functions become uncoupled from inflammatory signaling (Magnani et al., 2020). The host may therefore exhibit simultaneous hyperinflammation and impaired antimicrobial competence. This paradox is a defining feature of septic immune dysfunction. Excessive inflammatory signaling continues to damage tissues, while the actual capacity to clear infection and restore homeostasis weakens.

Mitochondrial dysfunction also contributes to immune exhaustion and apoptotic loss of immune cells, further reducing host resilience (Angajala et al., 2018). In this context, immune failure is not simply a consequence of pathogen burden or immune suppression imposed from outside the cell; it is a bioenergetic defect within the immune system itself. This makes mitochondrial integrity a central determinant of whether the host response remains protective or collapses into dysfunctional persistence.

### Cerebral vulnerability: mitochondrial dysfunction in sepsis-associated encephalopathy

The brain, which consumes approximately 20% of total resting energy expenditure and relies almost exclusively on aerobic oxidative phosphorylation for ATP synthesis, is exquisitely vulnerable to mitochondrial dysfunction during sepsis (Preau et al., 2021). This vulnerability is clinically manifested as sepsis-associated encephalopathy (SAE), a diffuse cerebral dysfunction that affects approximately 50–70% of patients with severe sepsis and ranges from mild disorientation and delirium to stupor and coma, with long-term cognitive impairment persisting in a substantial proportion of survivors (Zhao et al., 2024).

Mitochondrial injury in the septic brain mirrors the core mechanisms described in parenchymal organs but occurs with distinctive temporal and cellular features. Preclinical models have demonstrated that sepsis induces widespread mitochondrial perturbations in cerebral tissue, encompassing alterations in mitochondrial morphology, respiratory chain dysfunction, and escalation of reactive oxygen species, which collectively undermine neuronal bioenergetics and contribute to neurodegeneration. At the molecular level, sepsis induces a significant downregulation of mitochondrial quality-control proteins in brain tissue, including Pyruvate Kinase M2 (PKM2) and Prohibitin 2 (PHB2), with corresponding impairments in mitochondrial dynamics—including fission, fusion, and mitophagy. Overexpression of PKM2 and PHB2 not only restores mitochondrial function, as evidenced by normalized ATP production and membrane potential, but also confers resistance to oxidative stress by mitigating reactive oxygen species generation and protects against sepsis-induced neuronal apoptosis. These findings highlight the centrality of mitochondrial quality control in neuronal survival during sepsis and identify the PKM2-PHB2 interaction as a potential therapeutic target for septic cerebral injury (Zhao et al., 2024).

Pyruvate metabolism, a key intersection between glycolysis and oxidative phosphorylation, is particularly disrupted in the septic brain. Pyruvate dehydrogenase kinase 4 (PDK4), which inhibits pyruvate dehydrogenase complex activity through phosphorylation, is markedly elevated in septic states and has been identified as a critical regulator in SAE pathogenesis. Single-cell analysis has demonstrated that PDK4 is closely associated with microglia activation in SAE, and its expression is upregulated in the hippocampus of septic mice. Mechanistically, PDK4-mediated suppression of pyruvate oxidation drives microglial pyroptosis through ROS-dependent NLRP3 inflammasome activation, establishing a mechanistic link between mitochondrial metabolic reprogramming and neuroinflammation. This pathway is therapeutically targetable: dichloroacetate, a pyruvate dehydrogenase kinase inhibitor, has been shown to improve mitochondrial membrane potential, reduce reactive oxygen species generation, inhibit NLRP3-mediated microglial pyroptosis, reduce neuronal death, and rescue cognitive function in LPS-induced SAE models (Huang et al., 2025). These findings align with the broader framework of PDC-targeted nutritional strategies discussed in this review and suggest that thiamine—an essential PDC cofactor—may have particular relevance in cerebral bioenergetic rescue.

The recognition of cerebral mitochondrial vulnerability has direct therapeutic implications. The brain’s strict dependence on oxidative phosphorylation and its susceptibility to substrate limitation mean that interventions targeting pyruvate dehydrogenase activity (thiamine), mitochondrial substrate trafficking (carnitine), and electron transport chain stability (coenzyme Q10) may hold particular promise for preventing or ameliorating SAE. Moreover, emerging evidence for mitochondrial-targeted interventions—including mitophagy activators, mitochondrial biogenesis modifiers, and antioxidants that preserve mitochondrial membrane potential—in improving cognitive function in preclinical SAE models suggests that the nutritional strategies evaluated in this review could be rationally extended to cerebral protection (Zhang et al., 2025). However, the blood–brain barrier presents a unique pharmacokinetic challenge that must be addressed in future translational studies, as systemic nutrient delivery may not achieve adequate central nervous system concentrations without optimized dosing or adjunctive strategies.

### Endothelial dysfunction, microcirculatory failure and tissue hyporesponsiveness

Sepsis related mitochondrial injury extends beyond immune cells to the vascular interface, where endothelial dysfunction contributes substantially to organ hyporesponsiveness (Sygitowicz and Sitkiewicz, 2020). Endothelial cells are often described as regulators of vascular tone and barrier function, but in sepsis they are also metabolically stressed signaling cells whose dysfunction can destabilize perfusion even when macrocirculatory targets appear acceptable. Mitochondrial impairment in the endothelium alters redox signaling, barrier integrity, vasoregulatory responsiveness and interaction with inflammatory mediators, thereby weakening microvascular coordination (Kirkman et al., 2021).

The consequence is microcirculatory failure in a functional rather than purely structural sense. Tissue beds may continue to receive blood flow, yet extraction, distribution and local responsiveness become increasingly heterogeneous (Xantus et al., 2021). Capillary flow becomes less effectively matched to cellular demand, endothelial permeability rises and inflammatory activation further impairs microvascular efficiency (Raia and Zafrani, 2022). In this setting, tissue hypoxia and energetic insufficiency may persist despite normalization of blood pressure or oxygen content because the interface between circulation and cellular utilization has become metabolically unstable.

Tissue hyporesponsiveness emerges from this mismatch. Cells exposed to inconsistent microvascular support and ongoing inflammatory stress become less capable of mounting efficient metabolic responses (Joffre and Hellman, 2021). This does not only reflect poor perfusion; it reflects a failure of vascular and parenchymal bioenergetics to operate as an integrated system. Endothelial mitochondrial dysfunction is therefore central to the septic phenotype in which organs appear perfused at the systemic level but remain functionally compromised at the tissue level (Pang et al., 2024).

### Mitochondrial injury in cardiac, renal, hepatic and skeletal muscle dysfunction

The organ level consequences of septic mitochondrial dysfunction are particularly evident in tissues with high energetic requirements. In the heart, mitochondrial injury contributes to impaired contractile performance, reduced energetic reserve and diminished responsiveness to stress (Morciano et al., 2021). Septic myocardial dysfunction is not explained solely by circulatory factors or inflammatory mediators acting at the cell surface; it is also driven by the inability of cardiomyocytes to sustain oxidative metabolism and ATP production under inflammatory pressure (Wasyluk et al., 2021).

Mitochondrial function is critical for maintaining structure (integrity), solute transport across membranes and gradient stability in tubular epithelial cells of the kidneys. When there is a loss of function in mitochondria within these cell types, it can result in a decrease in cellular ability to perform its functions as well as an increase in cellular stress which progresses from adaptive downregulation to clinically relevant acute kidney injury (Wang et al., 2024). Likewise, in the liver, mitochondrial impairment disrupts metabolic integration, biosynthetic capacity, detoxification processes and stress tolerance (LeFort et al., 2024). Hepatic dysfunction in sepsis is therefore both inflammatory and bioenergetic in nature.

Skeletal muscle provides another important example. Mitochondrial injury in muscle contributes to weakness, reduced respiratory muscle efficiency, delayed recovery and prolonged catabolic vulnerability (Chen et al., 2023). In critically ill patients, this has consequences that extend beyond mobility alone. Respiratory muscle weakness can worsen ventilatory dependence and secretion clearance, while generalized bioenergetic failure contributes to delayed rehabilitation and prolonged intensive care burden (Mankowski et al., 2021).

These organ manifestations are mechanistically linked by a common principle: mitochondrial injury lowers the threshold at which physiological stress becomes functional failure. The specific phenotype differs across tissues, but the underlying logic remains consistent. When cells cannot generate or deploy energy efficiently, organ work declines, repair slows and susceptibility to secondary injury rises.

### Crosstalk among inflammation, cell death and bioenergetic collapse

Mitochondrial dysfunction in sepsis does not operate in isolation from inflammation and cell death pathways; it is embedded in a dense network of reciprocal amplification (Hong et al., 2024). Inflammatory signaling promotes mitochondrial injury by oxidative stress, nitric oxide imbalance, altered calcium handling and enzyme inhibition. Injured mitochondria, in turn, release danger associated signals, amplify redox instability and sustain inflammatory activation (Itagaki et al., 2021). This reciprocal loop helps explain why septic injury often becomes self-propagating even after the initiating infection is brought under partial control.

Cell death adds another layer to this process. Bioenergetic failure can lead to apoptotic and necrotic pathways, while excessive or poorly regulated cell death further destabilizes tissues and impairs recovery (Zhang et al., 2025). In immune compartments, apoptotic loss contributes to host-defense failure. In the vasculature, endothelial death disrupts barrier function and microcirculatory coordination (Pang et al., 2024). Thus, inflammation, mitochondrial dysfunction and cell death are not sequential events but mutually reinforcing processes.

What is especially important is that this crosstalk alters the trajectory of sepsis. A cell exposed to transient inflammation may recover if mitochondrial function remains intact and quality-control pathways are effective. By contrast, when inflammation is accompanied by persistent mitochondrial injury and dysregulated cell death signaling, the same cell may cross into irreversible dysfunction. This helps explain why septic deterioration can be nonlinear and why patients may worsen despite partial systemic stabilization. The decisive event is often not the persistence of inflammation alone, but the failure of mitochondrial systems to contain its intracellular consequences.

This reciprocal amplification is further propagated by mitochondrial damage-associated molecular patterns (mtDAMPs). Under septic stress, excessive mtROS compromises mitochondrial membrane integrity, leading to the release of mitochondrial DNA (mtDNA), cardiolipin, and N-formyl peptides into the cytosol and circulation. These mtDAMPs are recognized by pattern recognition receptors including Toll-like receptor 9 (TLR9), NLRP3, and cGAS-STING, triggering robust pro-inflammatory cytokine production. Notably, mtDNA and mtROS synergistically promote NLRP3 inflammasome assembly, creating a feed-forward loop wherein mitochondrial injury sustains systemic inflammation and further organelle damage. Circulating cell-free mtDNA has emerged as a promising biomarker of mitochondrial injury in sepsis, correlating with disease severity and adverse outcomes. This DAMP-mediated amplification reinforces the rationale for mitochondrial-targeted strategies aimed at limiting both bioenergetic failure and DAMP-driven inflammatory propagation (Bushra et al., 2024; Lai et al., 2025).

### Mitochondrial dysfunction as a systems-level driver of multiple organ failure

Taken together, these pathophysiological consequences establish mitochondrial dysfunction as a systems-level driver of multiple organ failure in sepsis. The heart, kidney, liver, endothelium, skeletal muscle and immune system do not fail independently; they fail within an interconnected biological landscape shaped by shared bioenergetic collapse (Srdić et al., 2024). Mitochondrial injury weakens ATP production, disrupts redox homeostasis, impairs quality control and reduces metabolic flexibility across cell types. These defects then manifest as immune paralysis, endothelial instability, tissue hyporesponsiveness, organ specific dysfunction and delayed recovery.

This systems perspective is essential because it reframes septic organ failure as more than the cumulative effect of isolated insults. It suggests that multiple organs enter a common state of reduced energetic competence, each expressing that state according to its functional demands and cellular architecture (FCCM, 2024). The immune system becomes ineffective, the microcirculation becomes incoherent, parenchymal tissues become metabolically restricted and recovery pathways lose efficiency (Liu et al., 2024). As this distributed failure progresses, supportive care can maintain life temporarily, but restoration of function becomes increasingly difficult unless mitochondrial homeostasis begins to recover.

The concept of multiple organ failure as a bioenergetic syndrome also strengthens the therapeutic case for metabolic and mitochondrial rescue (Yap et al., 2025). If mitochondrial dysfunction is upstream of diverse organ phenotypes, then interventions that stabilize mitochondrial function may have broader consequences than those targeting a single inflammatory mediator or organ system alone. This does not diminish the importance of infection control and conventional critical care, but it highlights why organ support without intracellular recovery may be insufficient.

Before turning to nutrient-based rescue strategies, it is useful to visualize how mitochondrial dysfunction propagates across immune, endothelial and parenchymal compartments to produce septic organ failure. The figure below depicts immune-cell metabolic paralysis, endothelial and microcirculatory dysfunction, parenchymal energetic failure, inflammatory amplification and systems-level progression to multiple organ dysfunction (Srdić et al., 2024).

## Nutritional strategies for restoring bioenergetic homeostasis

### Thiamine and reactivation of pyruvate dehydrogenase-dependent metabolism

Among nutrient-based mitochondrial interventions in sepsis, thiamine occupies a particularly strong mechanistic position because it directly supports one of the most vulnerable metabolic nodes in septic bioenergetic failure: pyruvate dehydrogenase complex activity (Moskowitz and Donnino, 2020). As an essential cofactor for pyruvate dehydrogenase, thiamine is required for efficient conversion of pyruvate into acetyl-CoA and for subsequent entry of carbon substrates into the tricarboxylic acid cycle (Velazquez-Arellano and Hernandez-Vazquez, 2020). In sepsis, where pyruvate dehydrogenase activity is often functionally suppressed, thiamine becomes relevant not merely as a vitamin, but as a metabolic gatekeeper.

The therapeutic logic is straightforward but biologically important. When pyruvate cannot be directed efficiently into oxidative metabolism, cells increasingly depend on metabolically inefficient pathways, lactate handling deteriorates and ATP-generating capacity falls. Thiamine supplementation may help relieve this bottleneck by facilitating oxidative substrate flux, improving mitochondrial use of glycolytic products and reducing the mismatch between substrate availability and mitochondrial utilization (Ritorto et al., 2025). This is especially relevant in tissues operating near energetic limits, including myocardium, kidney, liver and activated immune cells. Human evidence for thiamine in sepsis remains limited to small clinical trials and observational studies; no large‑scale randomized controlled trial has definitively established clinical benefit. The therapeutic rationale presented here is therefore primarily mechanism‑based, with human data serving as supportive but not conclusive.

Several randomized controlled trials have evaluated thiamine in septic populations. A 2026 meta-analysis of 9 RCTs reported that thiamine significantly reduced renal replacement therapy requirements and improved lactate clearance and SOFA scores, but short-term mortality was not significantly reduced. In thiamine-deficient patients, an exploratory subgroup analysis showed a potential mortality benefit (Xu et al., 2026). A 2025 meta-analysis of 6 RCTs confirmed reduced in-hospital mortality and RRT requirements in septic shock patients receiving thiamine (Wang et al., 2026). A large retrospective study using the MIMIC-IV database found that thiamine non-use was associated with increased hospital mortality and ICU mortality (Yin et al., 2026). Additionally, a randomized trial of hydrocortisone-thiamine-vitamin C triple therapy reported lower hospital mortality in the treatment group (Jamshidi et al., 2021). These findings require prospective validation before any clinical recommendation can be made.

### Carnitine and restoration of mitochondrial substrate trafficking and fatty acid oxidation

If thiamine primarily addresses oxidative entry of carbohydrate-derived substrates, carnitine addresses a different but complementary component of bioenergetic homeostasis: mitochondrial substrate trafficking. Carnitine is essential for the transport of long-chain fatty acids into mitochondria for β-oxidation, thereby influencing substrate flexibility, energy generation and metabolic balance under stress (Keshani et al., 2022). In sepsis, where fuel use becomes disordered and mitochondrial efficiency declines, the ability to traffic substrates appropriately becomes increasingly important (Sahebnasagh et al., 2022).

The importance of carnitine is likely due to its ability to protect (or possibly) reestablish a cell’s adaptive use of available energy substrates as needed. Cells are able to shift from one source of energy to another based on cellular needs and the availability of energy substrates and oxidative capabilities within their mitochondria (Friedrich et al., 2021). In septic individuals, however, it appears that many cells lose the ability to make these shifts and instead appear to be stuck using the most available (but least efficient) pathways for energy production and accumulate intermediate products which indicate an incomplete process (Levy, 2007). Carnitine may help counter this state by supporting the controlled movement of fatty acid substrates into mitochondria and limiting metabolic stagnation associated with impaired substrate processing (Eaton and Pierro, 2005).

However, the translational significance of carnitine depends on context. Its potential benefit is most compelling where impaired mitochondrial substrate trafficking is a meaningful contributor to organ dysfunction (Jones et al., 2018). This suggests that carnitine should not be viewed as a generic metabolic enhancer, but as a targeted intervention most relevant to septic phenotypes characterized by disrupted fatty acid utilization, reduced metabolic flexibility, or evidence of mitochondrial fuel mismatch. While the RACE trial and other human studies provide some clinical data, the overall evidence base for carnitine in septic patients is modest. Preclinical studies strongly support its mechanistic role, but translation to routine clinical use requires further validation.

We have now further clarified that the largely neutral RACE trial does not refute carnitine’s mechanistic potential but rather underscores the need for biomarker-guided patient selection (Jones et al., 2018). A 2024 meta-analysis confirmed no significant mortality association overall but noted significant heterogeneity (I²=70%), suggesting that patient severity and timing may influence outcomes (Meng et al., 2024). Notably, pharmacometabolomics analysis of the RACE trial demonstrated that patients with baseline acetylcarnitine >35 µM derived a significant mortality benefit from high-dose L-carnitine (Puskarich et al., 2015). A recent RCT in 60 sepsis patients reported that L-carnitine 3 g/day for 7 days) significantly reduced 28-day mortality while improving inflammatory and oxidative stress markers (Keshani et al., 2022, Keshani et al., 2024). These findings suggest that L-carnitine may benefit specific metabolically defined subgroups, supporting its continued investigation as a targeted rather than universal therapy.

### Coenzyme Q10 and support of electron transport chain efficiency and redox balance

Coenzyme Q10 occupies a different but equally important position within the mitochondrial rescue framework because it directly supports electron transport chain function and redox stability (Donnino et al., 2015). As a key electron carrier within the inner mitochondrial membrane, coenzyme Q10 facilitates electron transfer between respiratory chain complexes and thereby contributes to efficient oxidative phosphorylation (Ali et al., 2021). In sepsis, where respiratory chain instability and excessive reactive oxygen species generation are central features of mitochondrial dysfunction, coenzyme Q10 has particular mechanistic appeal.

Its therapeutic rationale rests on two linked actions. First, by supporting electron transport, coenzyme Q10 may help improve respiratory efficiency and reduce electron leakage that contributes to mitochondrial reactive oxygen species production (Soltani et al., 2020). Second, through its redox-active properties, it may help buffer oxidative stress and limit damage to mitochondrial membranes and enzymes (Rocha et al., 2012). This dual relevance to both ATP production and oxidative control makes it especially attractive in septic states characterized by redox collapse.

This mechanistic positioning distinguishes coenzyme Q10 from many broader antioxidant strategies that have failed to achieve consistent clinical significance in sepsis. Its appeal lies in its more proximal relationship to mitochondrial function itself. Rather than scavenging oxidative products after damage has propagated, it may act closer to the site where respiratory inefficiency and oxidative excess converge. However, human studies of CoQ10 in sepsis are few and generally small; the mechanistic appeal is strong, but clinical evidence remains preliminary.

But recent evidence strengthens confidence that CoQ10 has clinically meaningful effects beyond biomarker changes, particularly regarding organ function outcomes. The 2020 trial demonstrated reduced in-hospital mortality and decreased TNF-α and MDA levels (Soltani et al., 2020). More substantially, the 2026 randomized controlled trial reported that CoQ10–210 mg/day significantly reduced vasopressor duration and mechanical ventilation duration, while also improving SOFA scores and inflammatory markers compared to standard care. However, 20% of CoQ10 patients experienced hypotension, representing a safety signal requiring attention (Eladly et al., 2026). A pilot trial of the related mitochondrial-targeted antioxidant MitoQ demonstrated significant oxidative biomarker improvement and vasoactive-inotropic score changes, but 28-day mortality did not differ significantly (Moradbaki et al., 2026). In contrast, the 2015 Donnino pilot trial found no clinical outcome differences despite successful plasma CoQ10 level elevation (Donnino et al., 2015). Given these conflicting results, we are moderately confident that CoQ10 improves organ function outcomes but cannot be highly confident regarding mortality benefit. We have explicitly cautioned that the current evidence base remains insufficient to recommend routine CoQ10 use, and larger blinded multicenter trials with safety monitoring are urgently needed.

### Integrated mechanistic framework: from mitochondrial lesions to nutrient-based rescue

The three core mechanistic lesions—PDC impairment, excessive mtROS generation, and defective mitophagy—do not operate in isolation but form a self-reinforcing network that propagates from subcellular dysfunction to systemic inflammation and organ injury (Hu et al., 2024). Within this framework, each nutritional molecule engages a distinct but complementary node: thiamine relieves the PDC bottleneck by supplying its essential cofactor thiamine pyrophosphate, as Nuyttens et al. demonstrated that TPP supplementation restores pyruvate oxidation and protects mice from sepsis lethality (Nuyttens et al., 2025); carnitine supports fatty acid trafficking and β-oxidation, preserving metabolic flexibility when carbohydrate oxidation is compromised; and coenzyme Q10 stabilizes electron transfer at complexes I and III, limiting electron leakage and mtROS generation, with clinical evidence showing significantly lower CoQ10 levels in septic shock patients correlated with inflammatory markers (Donnino et al., 2011). By targeting these interconnected lesions concurrently, these nutrients attenuate mitochondrial danger signal release, reduce inflammasome activation, and limit the endothelial and parenchymal injury that underlies septic multiple organ dysfunction. Thus, the conceptual pathway proceeds from molecular lesions through nutrient-based rescue to restored bioenergetics, reduced inflammation, and improved organ function—a framework that supports phenotype-sensitive metabolic intervention rather than indiscriminate supplementation. Notably, the mechanistic evidence supporting this framework is derived primarily from preclinical models—particularly the demonstration that TPP supplementation restores pyruvate oxidation in CLP mice and that CoQ10 levels correlate with inflammatory markers in septic patients—whereas direct clinical evidence for mortality benefit remains limited to subgroup analyses and small trials. This distinction underscores that the framework is mechanistically robust but clinically preliminary.

### Beyond single-nutrient strategies: emerging pharmacological modulators of mitochondrial metabolism

While thiamine, carnitine, and coenzyme Q10 represent mechanistically grounded nutritional approaches, the broader pharmacological landscape offers additional avenues. Among these, dichloroacetate (DCA) has attracted particular interest as a direct activator of pyruvate dehydrogenase complex (PDC) activity. DCA functions by inhibiting pyruvate dehydrogenase kinase (PDK), the enzyme responsible for phosphorylating and inactivating PDC, thereby shifting metabolism toward glucose oxidation and enhancing mitochondrial ATP production (Schoenmann et al., 2023). In a rat model of sepsis-associated encephalopathy (SAE), DCA administration (25–100 mg/kg) significantly improved survival and neurological outcomes, reduced neuroinflammation (TNF-α, IL-1β, IL-10), attenuated oxidative stress, and restored mitochondrial complex I activity and ATP levels through inhibition of Drp1-mediated mitochondrial fission (Wang et al., 2024). Similarly, in a murine sepsis model, DCA treatment restored cardiac PDC activity, improved stroke volume at 12 hours and contractility at 30 hours post-administration, with associated changes in metabolic intermediates (Smith et al., 2025).

Beyond PDC modulation, mitochondrial-targeted agents have demonstrated preclinical promise. SS-31 (elamipretide), which binds to the mitochondrial inner membrane, has been shown to preserve mitochondrial integrity and mitigate cognitive deficits in sepsis models by stabilizing membrane potential and reducing oxidative stress (Zhang et al., 2024). Mitoquinone (MitoQ), a mitochondria-targeted ubiquinone derivative, alleviated sepsis-induced acute lung injury in CLP mice through upregulation of citrate synthase, preservation of mitochondrial complex proteins, and suppression of mitochondrial ROS and apoptosis in pulmonary macrophages. Notably, MitoQ pretreatment significantly reduced lung injury scores and improved blood gas parameters in a dose-dependent manner (Sun et al., 2025).

Importantly, these agents are not mutually exclusive with nutritional strategies. DCA targets the same PDC bottleneck that thiamine supports as a cofactor, suggesting potential synergy. SS-31 and MitoQ address oxidative and membrane stability dimensions that CoQ10 also targets, but with enhanced mitochondrial specificity. However, the evidence remains predominantly preclinical, and rigorous clinical trials are needed before clinical use can be recommended.

### Toward biomarker-guided metabolic rescue in septic patients

An additional layer of complexity that underscores the necessity of biomarker-guided phenotyping is the longstanding “obesity paradox” in sepsis—the observation that overweight and obese patients often demonstrate improved survival compared to normal-weight counterparts, despite obesity being a well-established risk factor for numerous chronic diseases (Wang et al., 2026). While extensively documented, the mechanistic basis of this paradox remains contentious, with some attributing it to methodological biases such as BMI-based misclassification rather than genuine biological protection (Kartikasari et al., 2026). Emerging evidence suggests that adipose tissue adaptations may influence mitochondrial function during sepsis. In critical illness, white adipose tissue undergoes “browning,” characterized by increased mitochondrial density, UCP1 expression, and respiratory capacity, potentially representing a metabolically adaptive response to systemic stress (Alipoor et al., 2020; Petroni et al., 2022). However, experimental data paint a more complex picture: in endotoxemic mice, short-term obesity accelerated mortality and exacerbated cardiac inflammation while reducing maximum mitochondrial respiratory rate, despite increased fatty acid oxidation (Petroni et al., 2022). An international cohort study of critically ill patients with sepsis or intra-abdominal infection further demonstrated that BMI classification methods significantly influence the observed obesity paradox, highlighting the need for more refined metabolic phenotyping in sepsis outcomes research (Donckels et al., 2026). These observations suggest that obesity-induced alterations in fatty acid metabolism and mitochondrial bioenergetics may modulate sepsis outcomes in ways that are currently incompletely understood. Critically, this heterogeneity—whether driven by obesity phenotypes, disease phase, or other modifiers—reinforces the argument that generalized metabolic interventions are unlikely to succeed across all patients. Instead, the obesity paradox exemplifies why future studies must move beyond broad clinical categories and toward biomarker-guided stratification that accounts for individual variations in mitochondrial phenotype, metabolic reserve, and substrate utilization. Such an approach would enable more precise alignment of nutritional or pharmacological interventions with the dominant bioenergetic lesion in each patient.

The future of nutrient-based mitochondrial therapy in sepsis depends on moving from generalized supplementation toward biomarker guided metabolic rescue (Adrita, 2024). Sepsis is a clinically and biologically heterogeneous syndrome and mitochondrial dysfunction is unlikely to present in identical form across all patients (Hu et al., 2024). Some individuals may exhibit dominant impairment of pyruvate oxidation, others marked redox stress and others defective substrate flexibility or mitochondrial quality-control failure. In this setting, precision becomes more important than breadth.

A biomarker-guided approach would aim to identify the dominant energetic lesion and align therapy accordingly (Schuetz and Mueller, 2014). Markers of pyruvate dehydrogenase dysfunction, lactate handling, oxidative stress, mitochondrial injury, substrate utilization, or organ-specific energetic stress could help define which metabolic support strategy is most rational for a given patient (Gou et al., 2025). Such stratification would also improve timing. Nutritional rescue is likely to be most effective before mitochondrial injury becomes too advanced or too structurally fixed to respond meaningfully (Reitsema et al., 2019).

This precision framework underscores that nutritional molecules are most rationally deployed as part of a targeted strategy that integrates molecular diagnosis with therapeutic selection, rather than as undifferentiated adjuncts across all septic phenotypes. The table below summarizes the principal nutrient-based strategies discussed in this section, including their mitochondrial targets, mechanisms and translational roles in restoring bioenergetic homeostasis in sepsis. These three nutrients target complementary mitochondrial nodes and, when deployed in combination, may synergistically restore bioenergetic homeostasis, attenuate inflammatory amplification, and improve organ function in sepsis, as shown in **Table 3**.

**Table 3:** Nutritional molecules for restoring mitochondrial bioenergetic homeostasis in sepsis: targets, mechanisms and translational relevance.

Nutritional molecule	Primary mitochondrial target	Principal mechanism	Bioenergetic rationale in sepsis	Translational relevance
Thiamine (Moskowitz and Donnino, 2020)	Pyruvate dehydrogenase complex and oxidative carbohydrate metabolism	Supports conversion of pyruvate to acetyl-CoA and promotes tricarboxylic acid cycle entry	May relieve oxidative substrate bottleneck, reduce inefficient pyruvate diversion and improve ATP-generating efficiency	Most relevant in phenotypes marked by impaired pyruvate oxidation or functional thiamine insufficiency
Carnitine (Eaton and Pierro, 2005)	Mitochondrial substrate trafficking and fatty acid oxidation	Facilitates transport of long-chain fatty acids into mitochondria and supports acyl-group handling	May improve metabolic flexibility, reduce substrate stagnation and support energy production in high-demand tissues	Most relevant in phenotypes with impaired fatty acid utilization or metabolic inflexibility
Coenzyme Q10 (Soltani et al., 2020)	Electron transport chain and redox homeostasis	Supports electron transfer and helps stabilize oxidative phosphorylation while limiting redox imbalance	May improve respiratory efficiency, reduce electron leakage, preserve membrane potential and limit oxidative amplification	Most relevant in phenotypes marked by respiratory chain instability and oxidative stress
Combination or biomarker guided strategy (Adrita, 2024)	Multiple mitochondrial nodes	Aligns intervention with dominant bioenergetic lesion	May provide more coherent rescue of integrated mitochondrial dysfunction than non-selective supplementation	Highest potential in precision metabolic therapy frameworks

### Emerging mitochondrial-targeted nutritional therapies beyond thiamine, carnitine, and CoQ10

While thiamine, carnitine, and coenzyme Q10 represent mechanistically grounded interventions with direct translational evidence, the broader nutritional landscape offers additional molecules that target complementary aspects of mitochondrial dysfunction in sepsis. These emerging strategies, though at varying stages of preclinical development, underscore the expanding therapeutic potential of mitochondrial-directed nutrition.

Nicotiamide adenine dinucleotide (NAD^+^) precursors have attracted considerable interest, given that NAD^+^ depletion is a hallmark of bioenergetic failure in sepsis. β-Nicotinamide mononucleotide (NMN), a direct NAD^+^ precursor, has demonstrated protective effects in septic models. Cao et al. showed that NMN administration after sepsis onset elevated NAD^+^ levels, reduced oxidative stress and inflammation, attenuated multi-organ dysfunction, and improved survival in septic mice (Cao et al., 2023). Mechanistically, Ni et al. demonstrated that NMN protects septic hearts by preventing cyclophilin F modification and lysosomal dysfunction, thereby attenuating mitochondrial permeability transition pore opening and preserving ATP production (Ni et al., 2025). These findings position NAD^+^ repletion as a promising strategy for mitigating sepsis-induced mitochondrial dysfunction.

Resveratrol, a natural polyphenolic Sirt1 activator, has emerged as another mitochondrial-targeted nutritional molecule. Wang et al. demonstrated that resveratrol delivered via a self-assembling nanopeptide hydrogel enhanced Sirt1-mediated deacetylation of p62, promoting mitophagy and immunometabolic remodeling in macrophages, which mitigated sepsis-induced inflammation and reduced tissue damage (Wang et al., 2025). Mechanistically, resveratrol’s activation of the Sirt1-p62 axis suppresses pro-inflammatory responses and decreases mitochondrial ROS production.

Alpha-lipoic acid (ALA), a mitochondrial cofactor with potent antioxidant properties, has demonstrated protective effects in experimental endotoxemia. Vanasco et al. reported that ALA treatment prevented oxidative stress and mitochondrial dysfunction in LPS-treated rats, preserving complex I and IV activities while reducing nitric oxide production (Vanasco et al., 2008). Given that ALA is a cofactor for pyruvate dehydrogenase and α-ketoglutarate dehydrogenase, it may synergize with thiamine at the PDC level.

These emerging strategies—NAD^+^ precursors, resveratrol, and alpha-lipoic acid—target distinct but overlapping nodes within the mitochondrial damage network. Their preclinical promise supports the broader principle that mitochondrial-targeted nutritional therapy in sepsis should extend beyond the three established molecules, though rigorous clinical translation remains a critical next step.

## Translational challenges and limitations

Despite the mechanistic appeal of mitochondrial-targeted nutritional therapy in sepsis, several translational barriers limit its immediate clinical adoption. The first challenge is biological heterogeneity (Leligdowicz and Matthay, 2019). Sepsis is not a single metabolic state and mitochondrial dysfunction does not present uniformly across patients, organ systems, or disease phases (Biebelberg et al., 2024). Some patients may exhibit predominant pyruvate dehydrogenase impairment, others marked oxidative stress, others defective mitochondrial quality control and many a combination that evolves over time (Van Amstel et al., 2023). This heterogeneity makes generalized nutrient supplementation biologically imprecise and may partly explain why promising mechanistic interventions often yield inconsistent clinical results.

A second limitation lies in biomarker uncertainty. Although mitochondrial dysfunction is increasingly recognized as central to septic organ injury, clinically practical markers that accurately define mitochondrial phenotype, severity and reversibility remain limited (Zimmermann et al., 2025). An additional limitation specific to thiamine is the lack of routine thiamine level measurement in most ICUs, despite evidence that septic patients with occult thiamine deficiency may derive greater benefit. A *post hoc* analysis suggested that mortality reduction with thiamine was most pronounced in patients with baseline thiamine levels <150 nmol/L, highlighting the potential for biomarker-guided therapy. Until such stratification becomes standard, negative trial results in unselected populations may obscure genuine efficacy in deficient subgroups. Many available indicators are indirect, nonspecific, or difficult to interpret in the context of concurrent inflammation, shock, organ support and nutritional variability (Hung et al., 2020). Without reliable bedside tools to identify which mitochondrial lesion is dominant and when it is therapeutically modifiable, intervention remains largely empirical rather than precision-guided.

A further translational barrier is the lack of rapid, point-of-care availability for most mitochondrial nutritional biomarkers. For thiamine, while deficiency occurs in up to 20.5% of emergency sepsis patients, measurement requires HPLC or LC-MS/MS with turnaround times of hours to days, and no prospective trial has validated baseline thiamine levels as a predictor of treatment response (Miller et al., 2024; Vine et al., 2024). For carnitine, baseline acetylcarnitine >35 µM has been proposed as a predictive biomarker based on RACE trial *post hoc* analysis, but acylcarnitine profiling requires tandem mass spectrometry with turnaround exceeding 24–48 hours, which is incompatible with the early resuscitation window (Puskarich et al., 2021). For CoQ10, no established biomarker exists. Emerging point-of-care biosensor technology can detect PCT and IL-6 in under 25 minutes, but analogous platforms for thiamine or acylcarnitine remain undeveloped (Bouzada et al., 2026). Thus, biomarker-guided metabolic rescue remains a future aspiration requiring both prospective validation and bedside assay development.

Finally, implementation in critical care practice remains challenging. Even when a nutrient-based strategy is biologically rational, its translation depends on dose standardization, route of administration, interaction with existing therapies and integration into time-sensitive resuscitation workflows. Nutritional molecules are unlikely to improve outcomes merely by being available; they must be deployed within a framework that links mechanism, timing and phenotype. These limitations do not weaken the importance of mitochondrial dysfunction in sepsis. Rather, they define the conditions under which mitochondrial nutritional therapy must mature: from conceptually attractive supplementation to biomarker-informed, phenotype-sensitive metabolic intervention.

Additionally, it should be noted that this article is based on a narrative review and lacks a formal bias risk assessment. This review did not use structured bias assessment tools. The informal quality screening excluded some studies with clearly inadequate methods (see PRISMA flowchart), but did not systematically grade the strength of evidence. Therefore, readers should interpret the comprehensive evidence as hypothesis generation. Although this review integrates both preclinical and human data, it is important to emphasize that the number and quality of human interventional trials testing thiamine, carnitine, and CoQ10 in sepsis remain low. Most human studies are small, heterogeneous in design, and often lack robust mitochondrial outcome measures. Therefore, while the mechanistic framework is strongly supported by experimental evidence, clinical conclusions should be considered preliminary. We explicitly avoid making definitive recommendations for clinical practice and instead frame nutrient‑based strategies as a promising direction for future research.

## Future directions

A critical priority for future research is the translation of mechanistically promising preclinical findings into clinically validated interventions. While the evidence for PDC impairment, mtROS-driven inflammasome activation, and mitophagy failure is well-established in animal models and cell-based systems, human data remain sparse and largely observational. Few randomized controlled trials have tested thiamine, carnitine, or CoQ10 with robust mitochondrial endpoints, and those that exist are limited by small sample sizes, heterogeneous populations, and variable timing of intervention. Moving forward, trials must be designed with prespecified mitochondrial biomarkers—such as whole-blood thiamine levels, plasma acetylcarnitine profiles, or circulating mtDNA—to identify patients most likely to benefit, and should incorporate direct measures of mitochondrial function (e.g., oxygen consumption, membrane potential, or ATP synthesis) as secondary or exploratory endpoints. Additionally, the optimal timing, dosing, and combination strategies for these nutrients remain undefined; preclinical studies suggest that early intervention before irreversible organ damage occurs is critical, but this has not been systematically tested in humans. Addressing these gaps will require multicenter, biomarker-stratified trials that evaluate not only survival but also organ function recovery, mitochondrial bioenergetic restoration, and long-term functional outcomes.

A second priority is biomarker development. The field needs robust, clinically feasible indicators that can define mitochondrial injury in real time, stratify patients by bioenergetic vulnerability and monitor treatment response. For thiamine specifically, future trials should incorporate baseline thiamine level measurement and restrict enrollment to deficient patients, as the signal for mortality benefit has only appeared in this subgroup to date. Such markers must move beyond nonspecific inflammatory or metabolic readouts and capture the functional state of mitochondrial substrate use, oxidative stress and quality control. Without this layer of precision, nutrient-based mitochondrial therapy will remain conceptually attractive but operationally imprecise.

Translational studies must also become more mechanistically disciplined. Future clinical trials should not test thiamine, carnitine, or coenzyme Q10 as broadly interchangeable adjuncts. They should be designed around a clear metabolic hypothesis, a defined septic phenotype and biologically relevant endpoints. This will be especially important for determining optimal timing, dosing and combination strategies. The most informative next-generation trials will likely be those that match intervention to mitochondrial lesion rather than evaluating nutritional molecules in unselected septic populations. For thiamine, we consider whole blood or plasma thiamine level the most clinically ready biomarker, as patients with documented deficiency consistently derive greater benefit. For carnitine, baseline acetylcarnitine levels or acylcarnitine profiles represent promising candidates based on pharmacometabolomics analyses, though prospective validation is needed. For CoQ10, no specific biomarker has been established, and plasma CoQ10 levels remain a logical but unproven candidate. Collectively, these biomarker strategies have been incorporated into our revised manuscript to guide future trial design. Based on the evidence summarized in this review, we do not envision mitochondrial-directed nutrition as a universal strategy applicable to most septic patients. Rather, these interventions are likely to benefit specific phenotypes: patients with documented thiamine deficiency (who showed a mortality signal in subgroup analyses), those with specific bioenergetic signatures such as elevated baseline acetylcarnitine levels (who derived benefit from L-carnitine in *post hoc* analyses), and potentially children with high-risk sepsis phenotypes characterized by altered lysine, carnitine, and fatty acid metabolism. This phenotype-specific approach—rather than universal supplementation—should guide future trial design.

Another key direction is integration across scales of biology. Mitochondrial dysfunction in sepsis influences immune competence, endothelial regulation, parenchymal organ performance and recovery kinetics simultaneously. Future research should therefore connect molecular findings with systems-level consequences, including microcirculatory behavior, organ-support trajectories and recovery from critical illness. This integrated approach will be essential for determining whether mitochondrial rescue produces meaningful clinical benefit beyond biochemical improvement.

Finally, implementation in critical care practice remains challenging. Even when a nutrient-based strategy is biologically rational, its translation depends on dose standardization, route of administration, interaction with existing therapies and integration into time-sensitive resuscitation workflows. These limitations define the conditions under which mitochondrial nutritional therapy must mature: from conceptually attractive supplementation to biomarker-informed, phenotype-sensitive metabolic intervention.

## Conclusion

Mitochondrial dysfunction is increasingly recognized as a central mechanistic axis of sepsis rather than a secondary by-product of critical illness. The syndrome’s progression from dysregulated host response to multiple organ failure is marked by a loss of bioenergetic coordination across immune, endothelial, parenchymal, and cerebral compartments. Within this framework, pyruvate dehydrogenase complex impairment, excessive mitochondrial reactive oxygen species generation, and defective mitophagy emerge as especially important because they disrupt substrate utilization, amplify oxidative injury, and prevent effective mitochondrial recovery. Thiamine, carnitine, and coenzyme Q10 target distinct but complementary nodes within this integrated damage network, while emerging pharmacological agents such as dichloroacetate, SS-31, and MitoQ expand the therapeutic landscape. Their potential value lies not in broad supplementation alone, but in their capacity to address specific points of mitochondrial vulnerability. However, the field remains limited by biological heterogeneity, imperfect biomarkers, and inconsistent clinical translation. Mitochondrial dysfunction in sepsis is dynamic, multidimensional, and unlikely to respond uniformly to any single intervention. The next phase of progress will therefore require biomarker-guided phenotyping, better alignment of intervention timing with metabolic injury stage, and clinically grounded trials that test mitochondrial rescue within defined patient profiles. If future work can connect mechanistic precision with clinically usable metabolic stratification, mitochondrial-targeted nutritional therapy may become an important complement to standard sepsis care.

## Data Availability

The original contributions presented in the study are included in the article/supplementary material. Further inquiries can be directed to the corresponding authors.
